# Suppression of Gq Function Using Intra-Pipette Delivery of shRNA during Extracellular Recording in the Ventral Tegmental Area

**DOI:** 10.3389/fncel.2013.00007

**Published:** 2013-02-12

**Authors:** Sudarat Nimitvilai, Devinder S. Arora, Maureen A. McElvain, Mark S. Brodie

**Affiliations:** ^1^Department of Physiology and Biophysics, University of Illinois at ChicagoChicago, IL, USA

**Keywords:** desensitization, dopamine D2 receptor, ventral tegmental area, neurotensin, Gq, shRNA, lentivirus

## Abstract

Selective suppression of protein function in the brain can be achieved using specific silencing RNAs administered *in vivo*. A viral delivery system is often employed to transfect neurons with small hairpin RNA (shRNA) directed against specific proteins, and intervals of several days are allowed between microinjection of the shRNA-containing virus into the brain and experiments to assess suppression of gene function. Here we report studies using extracellular recording of dopaminergic neurons of the ventral tegmental area (DA VTA neurons) recorded in brain slices in which lentivirus containing shRNA directed against Gq was included in the recording pipette, and suppression of Gq-related function was observed within the time frame of the recording. The action of neurotensin (NT) is associated with activation of Gq, and the firing rate of DA VTA neurons is increased by NT. With shRNA directed against Gq in the pipette, there was a significant reduction of NT excitation within 2 h. Likewise, time-dependent dopamine desensitization, which we have hypothesized to be Gq-dependent, was not observed when shRNA directed against Gq was present in the pipette and dopamine was tested 2 h after initiation of recording. As the time interval (2 h) is relatively short, we tested whether blockade of protein synthesis with cycloheximide delivered via the recording pipette would alter Gq-linked responses similarly. Both NT-induced excitation and dopamine desensitization were inhibited in the presence of cycloheximide. Inclusion of shRNA in the recording pipette may be an efficient and selective way to dampen responses linked to Gq, and, more generally, the use of lentiviral-packaged shRNA in the recording pipette is a means to produce selective inhibition of the function of specific proteins in experiments.

## Introduction

In receptor-based pharmacology, specific antagonists are usually used to reduce receptor function to help understand the role of that receptor in physiological processes (Cooper et al., 1996). Other pharmacological agents can be used to interfere with intracellular processes, such as second messenger activation (e.g., diacylglycerol) or the subsequent actions of those second messengers on other intracellular signaling proteins (e.g., protein kinase C; Nishizuka, 2001). One aspect of the use of such agents is that they are often not specific enough to distinguish among protein isoforms. Currently, the most specific means to interfere with protein or neurotransmitter action is to alter the expression of a specific gene product. Knockout or knockin mice, for example, have been developed to assess the role of GABA receptor subtypes in the responses to alcohol and other agents (Borghese et al., 2006; Halonen et al., 2009).

Complications with developmental adaptation as a result of knockouts have led to the development of conditional and inducible knockouts, in which knockouts can be limited to a specific brain region (conditional) or treatment with agents such as certain antibiotics (inducible) lead to interference with transcription of specific gene products in specific brain areas or at a specific postnatal time. These methods are costly, as making gene modifications and breeding of appropriate subjects can require a lot of time and resources (e.g., animal housing and maintenance). In addition, efficiency of knockout of specific genes may vary depending on the gene locus and Cre activity in specific cells (Sauer, 1998). Acute delivery of small hairpin RNA (shRNA) can reduce gene expression selectively. This method employs short strands of RNA that contains a tight hairpin formation and that are designed to bind specifically to regions of the mRNA coding the protein of interest. Binding of the shRNA is sufficient to suppress translation of the protein (Burnett and Rossi, 2012). Because of the specificity of the shRNA sequence, this method can be much more selective than receptor-based pharmacological methods. Generally, the shRNA is packaged within a viral or bacterial vector to facilitate entry into the cell. Most published methods indicate that the time frame needed for adequate suppression of function is on the order of days (Gupta et al., 2004) and sometimes may not be long-lived (Davidson and Boudreau, 2007).

The actions of many neurotransmitters are mediated by G-protein coupled receptors; stimulation of neurotransmitter receptors results in activation of specific G-proteins, which in turn modify the activity of ion channels or intracellular enzymes like kinases. A variety of neurotransmitter receptors are linked to the activation of a specific G-protein, Gq, including neurotensin (NT), serotonin, and dopamine receptors. Activation of these receptors results in activation of Gq, which in turn can initiate the intracellular signaling cascade involving phosphatidylinositol and protein kinase C (Rashid et al., 2007a). The half-life of Gq in some preparations has been shown to be on the order of 3–4 h (Mitchell et al., 1993; Johansson et al., 2005). Specific pharmacological antagonists of intracellular components like Gq are often difficult to obtain or unavailable, so it is difficult to demonstrate the specific involvement of such intracellular factors in receptor-mediated processes. One agent that has been reported to inhibit Gq by selectively interfering with GDP/GTP exchange (YM-254890; Taniguchi et al., 2003; Takasaki et al., 2004) has limited availability. The goal of the present study was to suppress the expression of Gq by acute application of lentivirus containing shRNA against Gq. We have recently published a number of papers in which pharmacological agonists and antagonists were included in the extracellular recording pipette in order to alter cell function (Nimitvilai et al., 2012a,c). In the present study, we assessed the feasibility of delivering lentivirus containing shRNA directed against Gq to a brain slice preparation directly via the recording electrode during extracellular electrophysiological experiments, and measuring the responses to Gq-related pharmacological phenomena. This method had the advantage of being able to measure the time course over which we could observe any possible reduction of Gq-related function. For comparison, in similar experiments, we assessed the effect of blocking transcription of all proteins by the inclusion of cycloheximide in the recording pipette.

## Materials and Methods

### Animals

Male Fischer 344 (F344; 90–150 gm) used in these studies were obtained from Harlan Sprague-Dawley (Indianapolis, IN, USA). All rats were treated in strict accordance with the NIH Guide for the Care and Use of Laboratory Animals and all experimental methods were approved by the Animal Care Committee of the University of Illinois at Chicago.

### Preparation of brain slices

The technique for preparing brain slices containing the ventral tegmental area (VTA) has been described previously (Brodie et al., 1999). Following rapid removal of the brain, the tissue was blocked coronally to contain the VTA and substantia nigra. The tissue block was mounted in the vibratome and submerged. Coronal sections (400 μm thick) were cut in chilled cutting solution using a vibratome, and the slices were placed onto a mesh platform in the recording chamber. The slice was submerged in artificial cerebrospinal fluid (aCSF) maintained at a flow rate of 2 ml/min and a temperature of 35°C. The composition of the aCSF in these experiments was (in mM): NaCl 126, KCl 2.5, NaH_2_PO_4_ 1.24, CaCl_2_ 2.4, MgSO_4_ 1.3, NaHCO_3_ 26, glucose 11. The composition of the cutting solution in the vibratome was (in mM): KCl 2.5, CaCl_2_ 2.4, MgSO_4_ 1.3, NaHCO_3_ 26, glucose 11, and sucrose 220. Both solutions were saturated with 95% O_2_/5% CO_2_ (pH = 7.4). Equilibration time of at least 1 h was allowed after placement of tissue in the recording chamber before electrodes were placed in the tissue.

### Cell identification

The VTA was clearly visible in the fresh tissue as a gray area medial to the darker substantia nigra, and separated from the nigra by white matter. Recording electrodes were placed in the VTA under visual control. pDAergic neurons have been shown to have distinctive electrophysiological characteristics (Grace and Bunney, 1984; Lacey et al., 1989). Only those neurons which were anatomically located within the VTA and which conformed to the criteria for pDAergic neurons established in the literature and in this laboratory (Lacey et al., 1989; Mueller and Brodie, 1989) were studied. These criteria include broad action potentials (2.5 ms or greater, measured as the width of the bi- or tri-phasic waveform at the baseline), slow spontaneous firing rate (0.5–5 Hz), and a regular interspike interval. Cells were not tested with opiate agonists as has been done by other groups to further characterize and categorize VTA neurons (Margolis et al., 2006). It should be noted that some neurons with the characteristics we used to identify dopaminergic neurons of the ventral tegmental area (DA VTA neurons) may not, in fact, be DA-containing (Margolis et al., 2006), and therefore the DA VTA neurons are referred to as putative DA (pDAergic) VTA neurons. A recent study (Chieng et al., 2011) provides some confidence that neurons with the electrophysiological characteristics noted above are, in fact, dopamine-containing.

### Drug administration

When drugs were added to the aCSF, this was done by means of a calibrated infusion pump from stock solutions 100–1000 times the desired final concentrations. The addition of drug solutions to the aCSF was performed in such a way as to permit the drug solution to mix completely with aCSF before this mixture reached the recording chamber. Final concentrations were calculated from aCSF flow rate, pump infusion rate, and concentration of drug stock solution. The small volume chamber (about 300 μl) used in these studies permitted the rapid application and washout of drug solutions. Typically drugs reach equilibrium in the tissue after 2–3 min of application.

In most experiments described here, drugs and lentivirus were added to the microelectrode filling solution (0.9% NaCl, about 23 μl per pipette). To assure effective concentrations of drugs were delivered to the neurons of interest, we used concentrations higher than would be used with bath application; the relatively high resistance pipette permits a gradual diffusion into the brain slice, so the drugs are diluted to an unknown concentration. When both drug application methods were tested, this pipette-application method (using higher intra-pipette concentrations) has produced results comparable to the bath application method (data not shown), with the advantage of more localized drug application and reduced expense. Such local delivery of drugs through recording pipettes has been used by our lab and others (Pesavento et al., 2000; Nimitvilai et al., 2012a).

DA hydrochloride, NT, and most of the salts used to prepare the extracellular media were purchased from Sigma (St. Louis, MO, USA). Cycloheximide was purchased from Tocris (Ellisville, MO, USA). Lentiviral particles and shRNA incorporated into that lentivirus were purchased from Santa Cruz Biotechnology, Inc. (Santa Cruz, CA, USA). The lentiviral particles used in these studies contained three to five expression constructs each encoding target-specific 19–25 nt (plus hairpin) shRNA designed to knockdown gene expression. Specific particles that were used were Gαq shRNA (sc-45998-V) and Golf shRNA (sc-108047-V).

For the Gαq shRNA, the lentiviral particles contained a pool of three different shRNA plasmids (A, B, and C). The sequence information is (Note: all sequences are provided in 5′ → 3′ orientation):

sc-45998-VA:
Hairpin sequence:
GATCCGCTATCTGACTCTACCAAATTCAAGAGATTTGGTAGAGTCAGATAGCTTTTT
Corresponding siRNA sequences (sc-45998A):
  - Sense: GCUAUCUGACUCUACCAAAtt
  - Antisense: UUUGGUAGAGUCAGAUAGCtt
sc-45998-VB:
Hairpin sequence:
GATCCGCTTAGCGAATACGATCAATTCAAGAGATTGATCGTATTCGCTAAGCTTTTT
Corresponding siRNA sequences (sc-45998B):
  - Sense: GCUUAGCGAAUACGAUCAAtt
  - Antisense: UUGAUCGUAUUCGCUAAGCtt
sc-45998-VC:
Hairpin sequence:
GATCCGGAGTACAATCTGGTCTAATTCAAGAGATTAGACCAGATTGTACTCCTTTTT
Corresponding siRNA sequences (sc-45998C):
  - Sense: GGAGUACAAUCUGGUCUAAtt
  - Antisense: UUAGACCAGAUUGUACUCCtt

Each siRNA sequence targets mRNA accession NM_031036. The locations are nucleotides 456 (Strand A), 693 (Strand B), and 1062 (Strand C) of NM_031036.

For the Golf shRNA, the lentiviral particles contained a pool of three different shRNA plasmids (A, B, and C). The sequence information is (Note: all sequences are provided in 5′ → 3′ orientation):

sc-108047-VA:
Hairpin sequence:
GATCCGTACAGACCACAAGATGTATTCAAGAGATACATCTTGTGGTCTGTACTTTTT
Corresponding siRNA sequences (sc-108047A):
  - Sense: GUACAGACCACAAGAUGUAtt
  - Antisense: UACAUCUUGUGGUCUGUACtt
sc-108047-VB:
Hairpin sequence:
GATCCGTAGTATCTGCCCATCTTATTCAAGAGATAAGATGGGCAGATACTACTTTTT
Corresponding siRNA sequences (sc-108047B):
  - Sense: GUAGUAUCUGCCCAUCUUAtt
  - Antisense: UAAGAUGGGCAGAUACUACtt
sc-108047-VC:
Hairpin sequence:
GATCCCCAACCATCCCTTAGTTAATTCAAGAGATTAACTAAGGGATGGTTGGTTTTT
Corresponding siRNA sequences (sc-108047C):
  - Sense: CCAACCAUCCCUUAGUUAAtt
  - Antisense: UUAACUAAGGGAUGGUUGGtt

Each siRNA sequence targets mRNA accession XM_001060758. The locations are nucleotides 2682 (Strand A), 2842 (Strand B), and 3130 (Strand C) of XM_001060758.

### Extracellular recording

Extracellular recording was chosen for these studies as this method permits the recordings to be stable and of long duration and allows us to assess the effects of extended exposure (>2 h) to drugs. The limitation of only measuring spontaneous action potential frequency (rather than membrane potential or other electrophysiological parameters) is counterbalanced by the advantage of being able to determine the time course of drug actions and interactions. Extracellular recording electrodes were made from 1.5 mm diameter glass tubing with filament and were filled with 0.9% NaCl with the addition of drugs of interest or appropriate control vehicle. Tip resistance of the microelectrodes ranged from 2 to 4 MΩ. A Fintronics amplifier was used in conjunction with an IBM-PC-based data acquisition system (ADInstruments, Inc.). Offline analysis was used to calculate, display, and store the frequency of firing 1 min intervals. Additional software was used to calculate the firing rate over 5 s intervals. Firing rate was determined before and during drug application. Firing rate was calculated over 1 min intervals prior to administration of drugs and during the drug effect; peak drug-induced changes in firing rate were expressed as the percentage change from the control firing rate according to the formula [(FRD − FRC)/FRC] × 100, where FRD is the firing rate during the peak drug effect and FRC is the control firing rate. The change in firing rate thus is expressed as a percentage of the initial firing rate, which controls for small changes in firing rate which may occur over time. This formula was used to calculate both excitatory and inhibitory drug effects. Peak excitation was defined as the peak increase in firing rate produced by the drug (e.g., NT) greater than the pre-drug baseline. Inhibition was defined as the lowest firing rate below the pre-drug baseline. Inhibition reversal was observed as a statistically significant reduction in the inhibition.

### Data collection

For comparison of the time course of effects on firing rate, the data were normalized and averaged. Firing rates over 1 min intervals were calculated and normalized to the 1-min interval immediately prior to the DA administration. These normalized data were averaged by synchronizing the data to the DA administration period, and graphs of the averaged data were made.

### Statistical analysis

Averaged numerical values were expressed as the mean ± SEM. Differences in firing rate over 2 h exposure to pipette contents were assessed using a paired *t*-test. Differences in the concentration of DAergic agonists and in the percentage inhibition among the groups were assessed with one-way ANOVA, followed by Tukey *post hoc* comparison. The differences among firing rates during the long drug administration intervals in these studies was assessed with two-way repeated measures ANOVA, followed by Student–Newman–Keuls or Tukey *post hoc* comparisons when needed (Kenakin, 1987). Pooled data in the figures below are expressed as relative change in firing rate; this permits normalization of the data to the inhibitory effect of dopamine at the first time point (5 min), and reflects the statistical analysis, which compared the effects of dopamine at each time point over time. Statistical analyses were performed with Origin 8.5 (Originlab Corporation, Northampton, MA, USA) or Sigmaplot 12 (Systat Software, Inc., San Jose, CA, USA).

## Results

### VTA neuron characteristics

A total of 72 VTA neurons were examined. Their initial firing rate ranged from 0.73 to 4.1 Hz, with a mean of 2.08 ± 0.08 Hz. All neurons had regular firing rates, and those cells exposed to dopamine were inhibited by it. Neurons tested with NT were exposed to the contents of the recording pipette for at least 2 h and cells were tested with dopamine only after 2 h of exposure to the contents of the recording pipette. The mean change in firing rate produced by this 2-h exposure is shown in Table 1; there was no significant difference in firing rate from the baseline rate at the initiation of the recordings and the rate at the 2-h time point in any of the experimental protocols (paired *t*-test, *p* > 0.05 for all groups).

**Table 1:** **Change in baseline firing rate over 2 h of recording with micropipettes containing lentivirus and/or shRNA**.

Condition	Starting baseline (Hz)	Baseline at 2 h (Hz)	Change in firing rate over pre-treatment period (%)
Figure 1C			
Gq shRNA + neurotensin	2.81 ± 0.29	2.18 ± 0.31	−15.0 ± ± 9.3
Lentiviral control + neurotensin	2.32 ± 0.36	3.11 ± 0.43	14.8 ± 10.55
Figure 2D			
Lentiviral control + dopamine	1.96 ± 0.19	1.81 ± 0.11	−3.53 ± 6.73
Golf + dopamine	2.36 ± 0.24	2.36 ± 0.26	−0.9 ± 5.7
Gq shRNA + dopamine	1.59 ± 0.22	1.46 ± 0.17	6.39 ± 5.71
Figure 3E			
Cycloheximide + neurotensin	1.86 ± 0.43	2.09 ± 0.8	18.5 ± 12.36
1% DMSO + neurotensin	2.25 ± 0.31	1.9 ± 0.35	−17.59 ± 7.3
Figure 3F			
1% DMSO + dopamine	2.23 ± 0.12	2.17 ± 0.17	−2.19 ± 6.57
Cycloheximide + dopamine	2.47 ± 0.45	2.09 ± 0.36	−7.65 ± 3.4

In neurons tested with NT, the concentration of NT was adjusted so that the firing rate of the VTA neuron was increased by about 50% during the first 10 min of the recording; that same concentration of NT was re-applied to that cell throughout the experiment. In neurons tested for dopamine-induced desensitization, sensitivity to dopamine (0.5–10 μM) was adjusted for each neuron so that inhibition exceeded 50%, as inhibition that was less than 50% was not reliably reversed (Nimitvilai and Brodie, 2010). This method of adjusting the concentration of DAergic agonist controlled for differences in sensitivity between neurons, but also sometimes resulted in the mean concentrations of dopamine slightly differing between groups. Overall, for pDAergic VTA neurons from adult rats, the concentration of dopamine used was 5.6 ± 0.5 μM (*n* = 39) which produced a mean change in firing rate of −74.4 ± 2.7% after 5 min of exposure to dopamine. There were no significant differences in the concentration of DAergic agonists [one-way ANOVA, *F*_(4,34)_ = 1.94, *p* > 0.05] or in the percentage inhibition [one-way ANOVA, *F*_(4,34)_ = 1.08, *p* > 0.05] among the groups. Cells which did not return to at least 70% of their pre-DA firing rate during this washout were not used. One benefit of the extracellular recording method used in these studies is that long duration recordings can be made reliably; the average recording duration was 171.8 ± 5.8 min, with a range of 125–270 min.

### Neurotensin excitation is reduced by Gq shRNA

**Figure 1:**
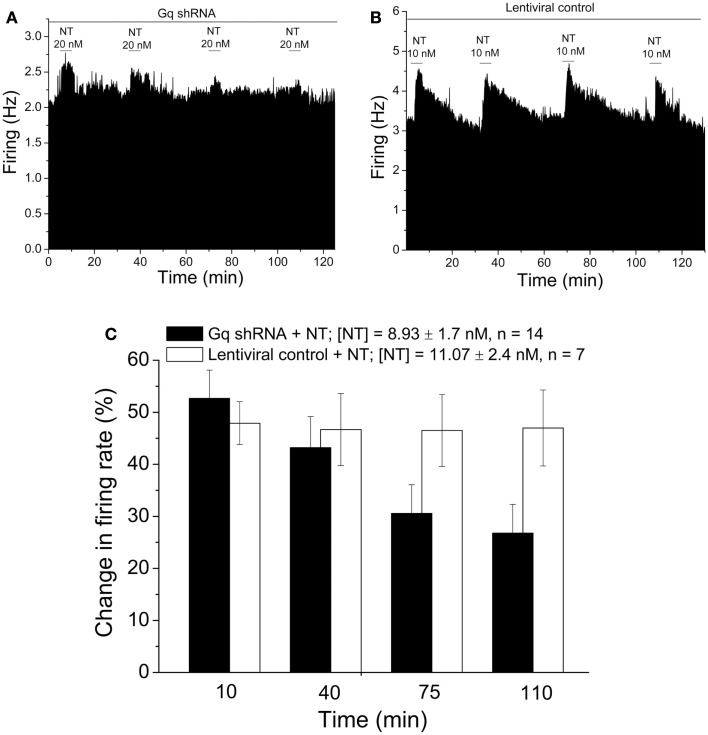
**Reduction of neurotensin-induced excitation of VTA neurons by Gq shRNA**. **(A,B)** Ratemeter diagrams. Firing rate over 5 s intervals is represented by the height of the vertical bars; duration of drug application is shown by the horizontal bars. Neurotensin (10 nM, 5 min applications) was tested at 30 min intervals while recording from DA VTA neurons with micropipettes containing 0.9% NaCl to which either lentivirus containing Gq shRNA **(A)** or lentivirus without shRNA **(B)** was added. The magnitude of neurotensin excitation over time in the recording shown in **(A)** was 25.99, 19.33, 8.3, and 2.7%, respectively; the magnitude of neurotensin excitation over time in the recording shown in **(B)** was 58.5, 62.8, 61.3, and 56.4%, respectively. **(C)** Bars representing the mean responses to neurotensin in recordings similar to those shown in **(A,B)**. Mean excitatory effect of neurotensin is shown at each time period for cells recorded with micropipettes to which Gq shRNA (filled bars) or lentiviral control (open bars) was added. There was a significant change in the excitatory effect of neurotensin in recordings in which Gq shRNA was present in the micropipettes.

Neurotensin is a major neurotransmitter in the VTA, and may play a role in regulating DAergic neurons during reward and reinforcement (Rompre et al., 1992). NT effects are mediated by activation of a Gq-linked receptor that is highly expressed in midbrain DAergic neurons (Palacios and Kuhar, 1981); NT increases firing rate of DAergic VTA neurons and dopamine release (Kalivas et al., 1983; Seutin et al., 1989; Farkas et al., 1997). We tested whether delivery of lentivirus containing shRNA directed against Gq via the recording pipette would alter NT-induced excitation of DA VTA neurons. Recordings were made with a pipette containing lentivirus containing shRNA directed against Gq (1 × 10^5^ IFU per 500 μl; about 23 μl per pipette). Five minutes after a recording of a DA VTA neuron was initiated, NT (a concentration sufficient to cause an increase in firing rate of approximately 50%, concentration from 5 to 50 nM, mean concentration 8.93 ± 1.7 nM) was added to the superfusate for 5 min and washed out for 30 min. The same concentration of NT was re-tested in this way at 30 min intervals. Typical results are shown in Figure 1A. It can be seen in this Figure that the effect of acute application of NT decreased over the time of the recording from 25.99% excitation to 2.7%. When lentivirus without shRNA was included in the recording pipette (1 × 10^5^ IFU per 500 μl; about 23 μl per pipette), there was no significant diminution of the magnitude of NT-induced excitation over time (Figure 1B). The mean results of experiments using pipettes with lentivirus (with and without shRNA) are shown in Figure 1C. Note that there was a significant interaction of shRNA and time [two-way repeated measures ANOVA, *F*_(3,83)_ = 7.69, *p* < 0.001], and there was a significant difference between Gq shRNA and control groups (Tukey *post hoc* for difference between 140 min time point vs. 10 min time point: *p* < 0.05). These results indicate that inclusion of lentivirus containing Gq shRNA in the recording pipette resulted in a reduction of the effect of NT to excite DA VTA neurons.

### DIR is suppressed by Gq shRNA

**Figure 2:**
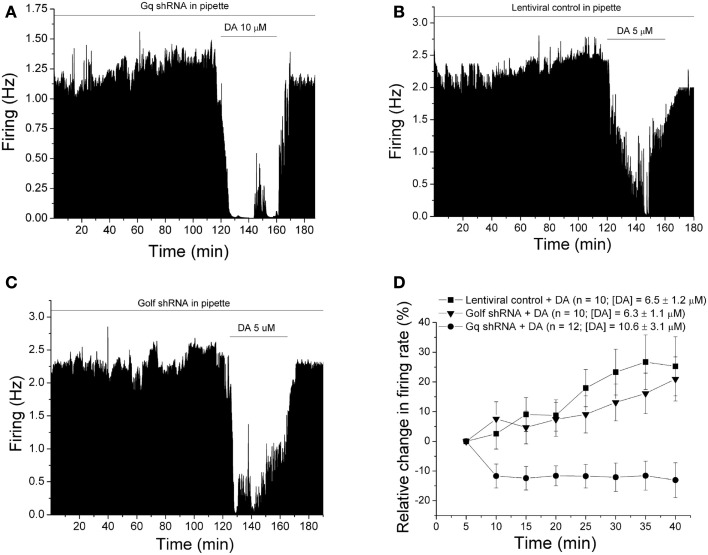
**Blockade of dopamine inhibition reversal by Gq shRNA**. **(A–C)** Ratemeter diagrams. Firing rate over 5 s intervals is represented by the height of the vertical bars; duration of drug application is shown by the horizontal bars. Recordings were maintained for 2 h before dopamine was applied for 40 min. Recordings were made from DA VTA neurons with micropipettes containing 0.9% NaCl to which either lentivirus containing Gq shRNA **(A)**, lentivirus without shRNA **(B)**, or lentivirus containing G_olf_ shRNA **(C)** was added. The inhibition produced by dopamine subsided over time when lentivirus without shRNA or lentivirus containing G_olf_ shRNA was present in the pipette. No reversal of dopamine-induced inhibition was observed when lentivirus containing Gq shRNA was in the micropipette. **(D)** Relative change in firing rate (mean ± SEM) is plotted as a function of time. In experiments similar to those shown in parts **(A–C)** above, the effect of dopamine at each time point was normalized by subtracting the change in firing rate (%) at the 5-min time point. There was a significant reversal in dopamine-induced inhibition when either Golf shRNA (▼) or lentiviral control (■) was present in the micropipettes. A significant inhibition produced by dopamine was observed with the inclusion of Gq shRNA (•) in the micropipettes.

We have recently published a number of papers characterizing the phenomenon of desensitization of responses to dopamine over time, a phenomenon which we have termed “dopamine inhibition reversal” (DIR; Nimitvilai and Brodie, 2010). We have shown that this phenomenon requires activation of both D2 and D1/D5 receptors (Nimitvilai and Brodie, 2010), is dependent on activation of phospholipase C and conventional protein kinase C pathway(Nimitvilai et al., 2012a,c), and is similar to a phenomenon reported to be linked to activation of Gq (Rashid et al., 2007b; So et al., 2009). In the present study, we tested whether inclusion of lentivirus with Gq shRNA could alter the development of DIR. One such experiment is shown in Figure 2A. After 2 h of recording with a micropipette containing Gq shRNA packaged in lentivirus (1 × 10^5^ IFU per 500 μl), we applied dopamine for 40 min; the inhibition produced by dopamine did not subside over time. In contrast, if lentivirus that did not contain shRNA was included in the micropipette (1 × 10^5^ IFU per 500 μl), we saw a clear reversal of the dopamine-induced inhibition over the 40-min time period of administration (Figure 2B) similar to the phenomenon of DIR that we have described previously (Nimitvilai and Brodie, 2010; Nimitvilai et al., 2012a,b,c, 2013). Likewise, if lentivirus containing shRNA directed against a different G-protein, Golf, was included in the pipette (1 × 10^5^ IFU per 500 μl), dopamine inhibition subsided over time (Figure 2C), again illustrating DIR that is observed under control conditions, and which we have described extensively previously. Golf shRNA was used instead of a scrambled shRNA control, and the lack of effect on DIR suggests that simply including shRNA in the pipette is insufficient to block DIR. The pooled data from experiments similar to these three examples is shown in Figure 2D. The magnitude of the dopamine response over time in comparison to the effect of dopamine at the 5-min time point is shown on this graph. A change in the positive direction indicates a decreasing effect of dopamine with time (DIR); a change in the negative direction indicates a greater inhibitory effect of dopamine with time. There was a significant difference in dopamine-induced inhibition with time [two-way repeated measures ANOVA, *F*_(12,223)_ = 3.24, *p* < 0.001]; Tukey *post hoc* comparison indicated for all times between 25 and 40 min that there was no difference (*p* > 0.05) between the Golf shRNA group and the control shRNA group, but there was a significant difference (*p* < 0.05) between the Gq shRNA group and both of the other groups (Figure 2D). These results indicate that DIR was blocked when Gq shRNA was included in the pipette, and not blocked by the inclusion of Golf shRNA.

### Both neurotensin-induced excitation and DIR are suppressed by cycloheximide

**Figure 3:**
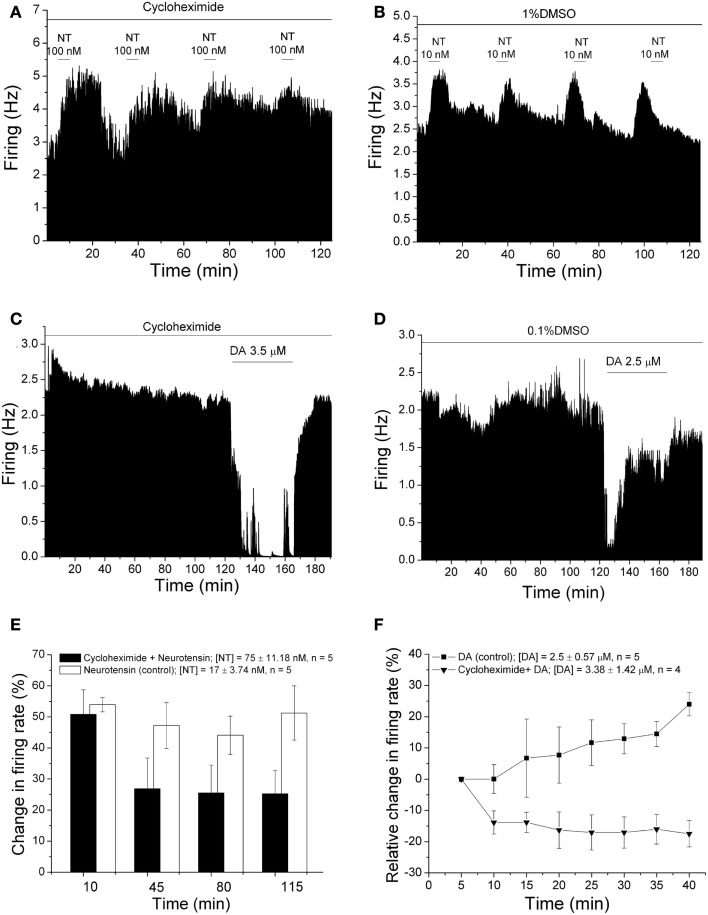
**Effect of cycloheximide on neurotensin excitation and DIR**. **(A–D)** Ratemeter diagrams. Firing rate over 5 s intervals is represented by the height of the vertical bars; duration of drug application is shown by the horizontal bars. **(A,B)** Neurotensin (100 nM) was applied for 5 min at 30 min intervals while recording from DA VTA neurons with micropipettes containing 0.9% NaCl to which either cycloheximide **(A)** or vehicle **(B)** was added. The magnitude of neurotensin excitation over time in the recording shown in **(A)** was 78, 17, 11.7, and 15%, respectively; the magnitude of neurotensin excitation over time in the recording shown in **(B)** was 55.4, 40.6, 43.2, and 52.8%, respectively. **(C,D)** Recordings were maintained for 2 h before dopamine was applied for 40 min. Recordings were made from DA VTA neurons with micropipettes containing 0.9% NaCl to which either cycloheximide **(C)** or vehicle **(D)** were added. The inhibition produced by dopamine subsided over time when vehicle was present in the pipette. No reversal of dopamine-induced inhibition was observed when cycloheximide was included in the micropipette. **(E)** Bars representing the mean responses to neurotensin in recordings similar to those shown in **(A,B)**. Mean excitatory effect of neurotensin is shown at each time period for cells recorded with micropipettes to which cycloheximide (filled bars) or vehicle (open bars) was added. There was a significant reduction in the excitatory effect of neurotensin in recordings in which cycloheximide was present in the micropipettes. **(F)** Relative change in firing rate (mean ± SEM) is plotted as a function of time. In experiments similar to those shown in **(C,D)** above, the effect of dopamine [after 2 h application of either cycloheximide (▼) or vehicle (■)] at each time point was normalized by subtracting the change in firing rate (%) at the 5-min time point. No reversal of dopamine-induced inhibition was observed when cycloheximide was present in the micropipettes.

If the changes observed with the lentiviral vector containing shRNA ultimately were due to decreased expression of Gq protein, suppression of overall protein synthesis should also produce a reduction in NT-induced excitation and DIR. While this does not necessarily confirm the efficacy of shRNA in the previous experiments, it is important to establish the fact that even non-specific suppression of protein synthesis can impair Gq-related functions. To test this hypothesis, we performed extracellular recordings with pipettes containing cycloheximide (800 μM) or 1% DMSO in 0.9% saline. Five minutes after initiating a recording, NT (concentration from 25 to 100 nM, mean concentration 75 ± 11.18 nM) was administered for 5 min and then again every 30 min for 2 h, to compare with the shRNA experiments (Figure 1). Examples of such experiments are shown in Figures 3A,B. In the presence of cycloheximide, acute application of NT initially increased the firing rate by 78.2%, and the effect of NT decreased over the time of the recording to 15.0% at 110 min (Figure 3A). Without cycloheximide, no diminution of the magnitude of NT-induced excitation was observed over time (Figure 3B). In the pool of recordings with cycloheximide-containing pipettes (Figure 3E), there was a significant difference between the cycloheximide and DMSO groups with an interaction of pipette-contents with time [two-way repeated measures ANOVA, *F*_(3,24)_ = 3.90, *p* < 0.03] and the Tukey *post hoc* test indicated that there was a significant difference between the groups at the 115 min time point (*p* < 0.05).

Similarly, we tested whether cycloheximide would prevent DIR as Gq shRNA had done in our lentivirus experiments. For these studies, we again included cycloheximide (800 μM) in the recording pipettes. Before testing DIR, a 120-min pre-incubation period was given to allow the cycloheximide to diffuse from the pipette into the surrounding tissue, as was done in the shRNA experiments. An example of an experiment in which this protocol was used is shown in Figure 3C. After the 120-min incubation period, dopamine (3.5 μM) was added to the superfusate, and administered for 40 min. No reduction in the inhibition produced by dopamine was apparent over the 40-min time course. However, a decrease in the inhibitory effect of dopamine was observed when cycloheximide was not present (Figure 3D), indicative of DIR. The pooled data from experiments similar to Figures 3C,D is shown in Figure 3F. There was a significant difference between the cycloheximide and DMSO groups with an interaction of pipette-contents with time [two-way repeated measures ANOVA, *F*_(7,49)_ = 7.79, *p* < 0.001] and Tukey *post hoc* test indicated that there was a significant difference between the groups at each time point from 15 to 40 min (*p* < 0.05). These results indicate that cycloheximide prevented the development of DIR.

## Discussion

Inclusion of agents in extracellular recording micropipettes is a method which uses small amounts of those agents and permits delivery to the vicinity of the neuron whose activity is being recorded. We have used this method with some success with a number of conventional pharmacological agents (Nimitvilai et al., 2012c). The results presented above indicate that inclusion of shRNA directed against Gq can reduce the effect of an agonist (NT) that is thought to produce excitation through activation of Gq (Seutin et al., 1989; Grisshammer and Hermans, 2001). In addition, we used this method to obtain information to support the idea that DIR is also mediated by Gq. While RNA and protein synthesis can take longer amounts of time, we found that there was sufficient time within a long recording period (2 h) to produce a significant effect on the response to NT. While we cannot estimate the actual degree of suppression of Gq in the region around the recording pipette, it has been reported that the half-life of degradation of Gq is short (about 3 h) in CHO cells with the addition of Gq-linked agonist. The effect of suppression of protein synthesis using a more general protein synthesis inhibitor (cycloheximide) supports the idea that suppression of protein synthesis (specifically or generally) can decrease electrophysiological responses to agonists within the time frame of an electrophysiological recording. General suppression of protein synthesis would be expected to have a variety of effects on cell function; what is interesting and relevant in this case is that suppression of Gq-related processes by cycloheximide was similar to inhibition of Gq processes following exposure to Gq shRNA. Our studies suggest that it may be possible to target certain proteins within cells very specifically and, in addition, to witness the time course of alteration of responses as a result of downregulation of expression induced by shRNA.

In support of this technique, it has been shown that delivery of double-stranded siRNA via a patch pipette in VTA neurons in brain slices could suppress the translation of GluR2 mRNA, resulting in a suppression of glutamate responses attributed to GluR2 activity (Mameli et al., 2007). The difference in the method used in the present report is the use of a lentiviral vector. As we did not have access to the intracellular compartment, the use of a carrier for moving the shRNA into the cell was required. Interestingly, in Mameli’s study, reduction of GluR2 was observed within 20 min, supporting the relatively rapid turnover of at least some proteins in VTA neurons maintained in brain slices.

Full suppression of NT-induced excitation was not achieved by any of these methods, suggesting a limit in the levels of suppression that could be achieved by blocking protein synthesis. Similarly, it has been reported in a pulse-chase study that there was a relatively rapid degradation of 60% of Gq, and that it took about 25 h to reduce Gq by an additional 20% (Johansson et al., 2005). Partial suppression of NT-induced excitation could reflect a partial reduction in Gq. On the other hand, DIR did appear to be completely suppressed. One factor could be that a portion of the NT activation of DA VTA neurons is not mediated by Gq, as the literature suggests that NT can inhibit (Gailly et al., 2000; Najimi et al., 2002) or stimulate (Yamada et al., 1993; Skrzydelski et al., 2003) adenylyl cyclase through its interaction with Gi and Gs, respectively, in some cell types. Alternatively, it may be that DIR is more sensitive to reduction of Gq than is NT-induced excitation, and may be completely disrupted by a partial suppression of Gq function.

One factor that should be noted in these experiments is the high multiplicity of infection (MOI) involved in these studies. The amount of vector within the pipette was approximately 4600 particles; we do not know what percentage of this load left the pipette to interact with the tissue. In addition, we have no way of knowing how many cells in addition to the neuron from which we are recording receive the lentivirus, but this method provides a relatively low-cost opportunity for a much higher MOI than would be achieved normally with intracranial injection of similar lentiviral particles. This high MOI could influence the efficiency with which the shRNA is delivered to the neuron, and may account for the fact that there was suppression of Gq-related processes within 2 h. Complex experiments may be necessary to reveal the degree of suppression of Gq, and the relationship between MOI and the results observed in the present experiments.

There may be many processes by which introduction of Gq shRNA may alter Gq mediated responses. Suppression of protein synthesis and reduction in incorporation of Gq protein into complexes involved in transduction of NT receptor stimulation is the most direct possibility (Burnett and Rossi, 2012). On the other hand, there may be regulatory processes within the neuron that are sensitive to shRNA (Bridge et al., 2003; Snove Jr. and Rossi, 2006), and may trigger other mechanisms of suppression of signal transduction that could occur more quickly than the processes involved in turnover of Gq protein. Alternatively, the Gq shRNA may have a relevant off-target effect, different from an off-target effect of Golf, that could interfere with DIR. Extensive additional studies will be needed to establish the degree of suppression of Gq under the conditions of the experiments described above, and whether that can fully account for the inhibition of NT-induced excitation and antagonism of DIR.

Similarly, we do not know that the shRNA affected the neurons from which we recorded specifically. Effects could have been due to actions on other neurons in the preparation, including GABergic neurons (Steffensen et al., 1998), or even glia within the brain slice. Extensive studies will be necessary to examine the spread of the shRNA and the specific cell types affected, and the relationship of those cells to the firing rate of pDAergic neurons of the VTA.

Here we present the method of inclusion of shRNA in a vector within the recording pipette in extracellular experiments; it offers a highly specific and useful way to examine the contribution of cellular factors to intracellular mechanisms. It is relatively inexpensive (as smaller amounts of lentiviral particles are required per experiment) and offers the opportunity to examine the time course of suppression of responses (as we did with NT-induced excitation).

The authors declare that the research was conducted in the absence of any commercial or financial relationships that could be construed as a potential conflict of interest.

## Conflict of Interest Statement

The authors declare that the research was conducted in the absence of any commercial or financial relationships that could be construed as a potential conflict of interest.
